# Orbital floor fractures: epidemiology and outcomes of 1594 reconstructions

**DOI:** 10.1007/s00068-021-01716-x

**Published:** 2021-06-14

**Authors:** Lukas Benedikt Seifert, Tim Mainka, Carlos Herrera-Vizcaino, Rene Verboket, Robert Sader

**Affiliations:** 1 Department of Oral, Cranio-Maxillofacial and Facial Plastic Surgery, University Hospital Frankfurt, Goethe University, Theodor-Stern-Kai 7, 60590 Frankfurt, Germany; 2 Department of Trauma-, Hand- and Reconstructive Surgery, University Hospital Frankfurt, Goethe University, Frankfurt, Germany

**Keywords:** Orbital fracture, Maxillofacial trauma, Maxillofacial reconstruction

## Abstract

**Objective:**

The aim of this study was to retrospectively review the midface and orbital floor fractures treated at our institution with regard to epidemiological aspects, surgical treatment options and postoperative complications and discuss this data with the current literature.

**Study design:**

One thousand five hundred and ninety-four patients with midface and orbital fractures treated at the Department of Oral, Cranio-Maxillofacial and Facial Plastic Surgery of the Goethe University Hospital in Frankfurt (Germany) between 2007 and 2017 were retrospectively reviewed. The patients were evaluated by age, gender, etiology, fracture pattern, defect size, surgical treatment and complications.

**Results:**

The average patient age was 46.2 (± 20.8). Most fractures (37.5%) occurred in the age between 16 and 35. Seventy-two percent of patients were male while 28% were female. The most common cause of injury was physical assault (32.0%) followed by falls (30.8%) and traffic accidents (17.0%). The average orbital wall defect size was 297.9 mm^2^ (± 190.8 mm2). For orbital floor reconstruction polydioxanone sheets (0.15 mm 38.3%, 0.25 mm 36.2%, 0.5 mm 2.8%) were mainly used, followed by titanium meshes (11.5%). Reconstructions with the 0.15 mm polydioxanone sheets showed the least complications (*p* < 0.01, *r* = 0.15). Eighteen percent of patients who showed persistent symptoms and post-operative complications: 12.9% suffered from persistent hypoesthesia, 4.4% suffered from post-operative diplopia and 3.9% showed intra-orbital hematoma.

**Conclusion:**

Results of the clinical outcome in our patients show that 0.15 mm resorbable polydioxanone sheets leads to significantly less post-operative complications for orbital floor defects even for defects beyond the recommended 200 mm^2^.

## Introduction

**Fig. 1:**
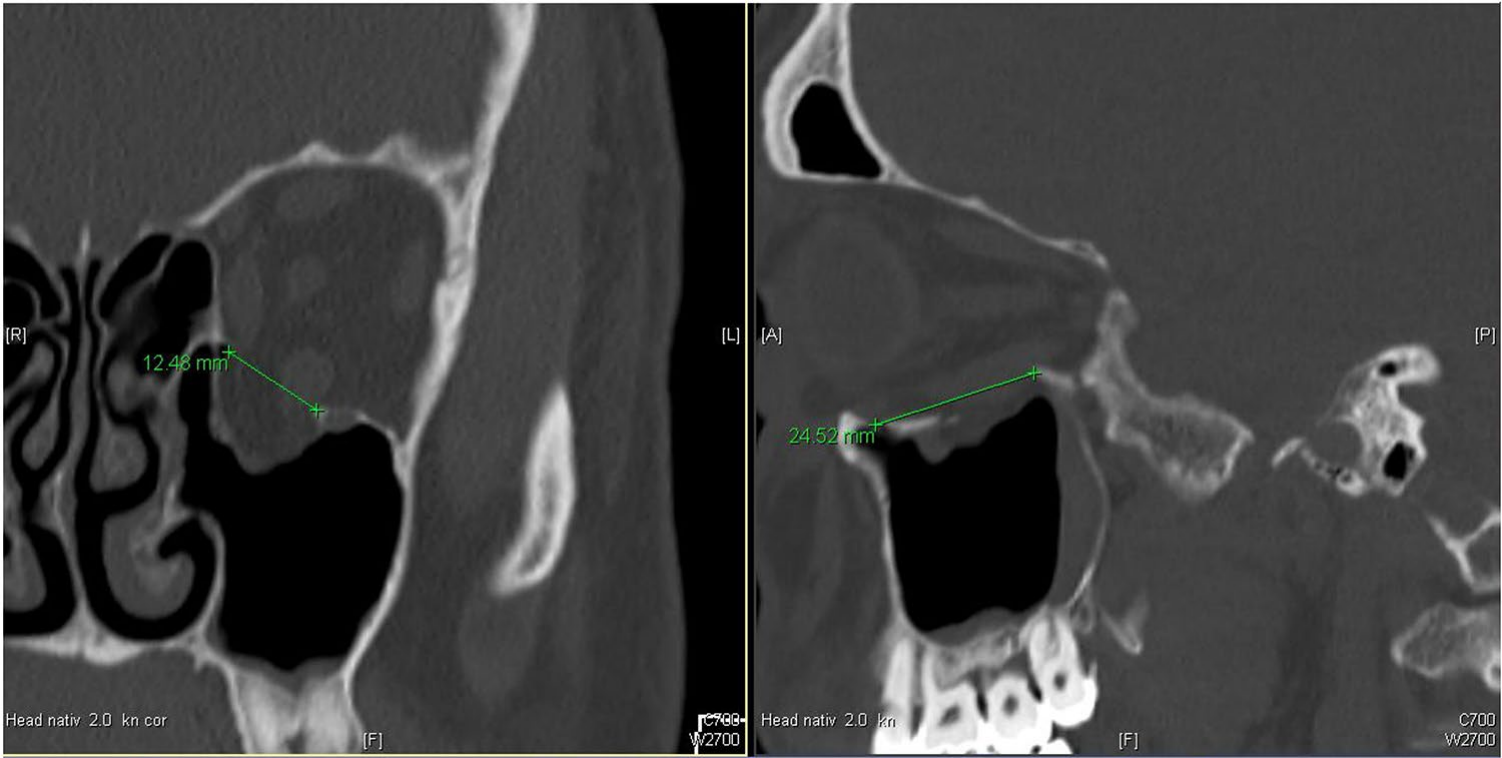
Coronal and sagittal CT slice of an isolated orbital floor fracture with a prolapse and incarceration of surrounding soft tissue. The size of the orbital floor defect was calculated by measuring the widest range of the defect in the coronal slices of the CT scans multiplied with the number of slices in which the defect was still visible and the layer thickness of the coronal slices

Epidemiological studies involving 600 hospitals in Germany from the TraumaNetzwerk DGU^®^ reported from a pooled number of patients of 102,887, that 11,451 (11.1%) suffered from facial trauma [1]. The orbit is especially susceptible to injury because of its very complex anatomical structure with hard and soft tissue. Blunt trauma in this facial region may cause an isolated orbital “blowout” fracture or a combined orbital fracture and midface fracture [2]. Today, there are a variety of classifications for midface fractures which include fractures of the orbit [3–5]. However, only the recently introduced AO classification for facial fractures permits a very precise description of the orbital injury pattern [5]. Depending on the type of fracture, patients can suffer from a variety of complications. Incarceration of the surrounding soft tissue (Fig. 1) can result in limitations of ocular movement [6], Diplopia [7] or enophthalmos. Another frequent complication is the irritation of the infraorbital nerve which runs in close proximity of the orbit and can result in temporary or permanent infraorbital hypoesthesia [8]. Furthermore, a retrobulbar hematoma can lead to a reduced or total loss of vision [9]. In addition to the clinical parameters, the computer tomography (Fig. 1) presents the gold standard to confirm the diagnosis [10].

The indication for a surgical intervention is controversially discussed throughout the last years. [11]. There is a general agreement that an acute loss of the visual function in the presence of a retrobulbar hematoma, severe enophthalmos, incarceration of peribulbar soft tissue and large defect sizes over 50% of the orbital floor require immediate surgical intervention to restore the anatomical structure of the orbit and improve the visual function as well as the orbital appearance [12–14].

**Fig. 2:**
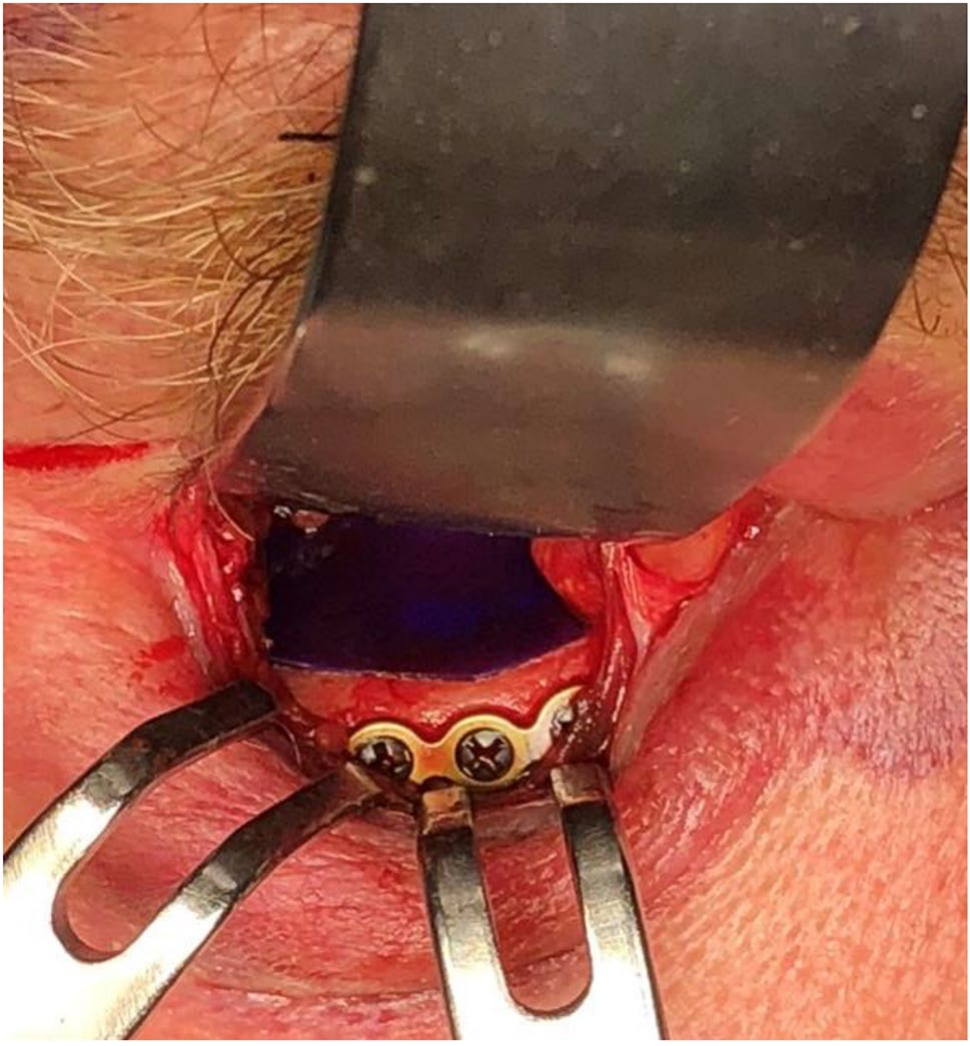
0.15 mm Poly-p-diaxanon (PDS^®^) in situ

**Fig. 3:**
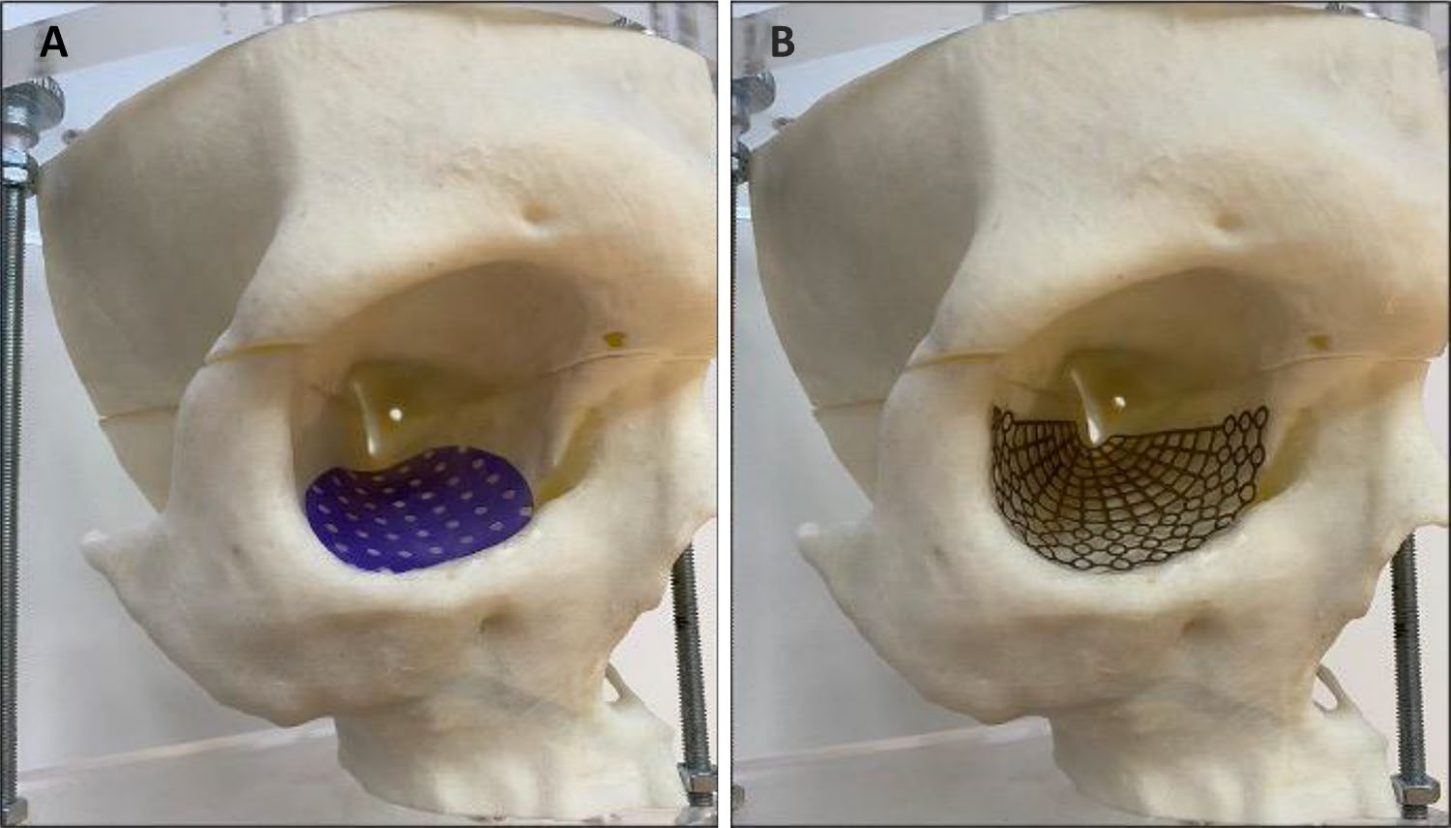
Personalized implanted biomaterial for orbital reconstruction. **A** Poly-p-diaxanon (PDS^®^) perforated membrane and **B** pre-bent titanium mesh

There are a variety of surgical approaches to reconstruct the orbit. The infraorbital, the subcilliary and the transconjunctival incision are commonly used whereby last is often recommended in the literature [15–18]. For a long time, autologous bone grafts were seen as the gold standard [19] to reconstruct the orbital floor. However, the unpredictable resorption rate as well as the missing optimal volume reconstruction led to the increasing use of resorbable alloplastic biomaterials, such as poly-p-dioxanon (PDS^®^) [20] (Fig. 2) and polyglactin (Ethisorb^®^) [21] and non-resorbable alloplastic materials, such as the titanium mesh (Fig. 3), which today can be individually preformed to the patients anatomy in a CAD-CAM process [22, 23].

There is a still a controversy about which material is suited best for orbital floor reconstruction regarding the post-operative outcome. In 2003, Ellis and Tan were the first to show that titanium meshes are more accurate in orbital reconstruction compared to bone grafts [24]. Other retrospective studies supported their results [25, 26]. Later Ellis and Messo estimated that titanium meshes are better suited to reconstruct the orbit compared to alloplastic materials, such as Teflon^®^ or PDS^®^ membranes, due to their incomplete degradation process which can result in sterile inflammation and development of granulomas [27]. On the other hand, it has been reported that titanium meshes cause inflammatory reactions [28] as well and are associated with significantly more post-operative complications, such as infections, extrusion and residual diplopia [29]. In 2002, Baumann et al. concluded that PDS^®^ membranes (0.25 mm or 0.5 mm) should only be used up to a defect size of 2.5 cm^2^ because of the high risk of developing enophthalmos due to the instability of the membrane [30]. Other authors extended this indication and could show that 0.25 mm PDS^®^ membranes were suitable even for larger defects (median 4.32 cm^2^) without significantly more complications, [20, 31] Unfortunately, none of the aforementioned studies included more than 78 patients which could be a limitation to their statistical power. To our knowledge, there is only one study that included more than 500 patients [32]. In their retrospective study, Holtmann et al. found that the use of 0.15 mm PDS^®^ membranes resulted in the best post-operative outcome for defect sizes between 250 and 300 mm^2^ [32].

The present literature is still divided regarding which material should preferably be used to reconstruct the orbit since no material only has advantages. Furthermore, it is unclear up to which defect size which material should be used. Therefore, the primary aim of this study was to retrospectively analyze different materials i.e. PDS^®^ membranes (0.15 mm; 0.25 mm; 0.5 mm) and titanium meshes in a large number of patients (1594) to evaluate which material could be recommended for which defect size. A secondary goal was to analyze and discuss the existing data with regard to epidemiological aspects, surgical treatment options and postoperative complications with the current literature.

## Methods

### Study design and methods

A retrospective study with a collective of 1874 patients with isolated or combined orbital fractures that were treated at the Department of Oral, Cranio-Maxillofacial and Facial Plastic Surgery, University Hospital Frankfurt, Germany in the years between 2007 and 2017 was conducted. Patients were diagnosed after clinical examination and radiological evaluation. Two hundred eighty (280) patients which were treated conservatively were excluded from the study, which resulted in 1594 included patients. Data was extracted from the internal patient management program “Orbis” from Agfa^®^ HealthCare GmbH (Bonn, Germany) and included pre- and post-operative radiological data, surgical documentation, follow-up clinical documentation, pre- and post-operative ophthalmological consultations. Target parameters included patient age and gender, time and causation of the accident, pre-operative clinical symptoms, such as hypoesthesia or diplopia, time between accident and treatment, type of fracture (isolated vs. combined), defect size, type of surgical incision, type of reconstruction material (0.15 mm, 0.25 mm, 0.5 mm polydioxanone sheets or titanium mesh) and the number of pre-, peri- and post-operative complications, such as retrobulbar hematoma, diplopia, hypoesthesia, visual impairment, displacement of the reconstruction material, infections and others. Typically, patients were followed up in the first four weeks after discharge and finally three months after discharge.

The size of the orbital floor defect was calculated according to Ellis and Ten [24] by measuring the widest range of the defect in the coronal slices of the CT scans multiplied with the number of slices in which the defect was still visible and the layer thickness of the coronal slices (Fig. 2).

The study was approved by the ethical board of the University Hospital Frankfurt with the trial registration number 391/18 and carried out under the regulations of the Declaration of Helsinki.

### Statistical analysis

Patient data were anonymized and transferred into Microsoft Excel^®^ (Microsoft Corporation, Redmond WA, USA, Version 16.35 for Mac). Statistical analysis was carried out using GraphPad Prism 8 for Mac, Version 8.4.2 (San Diego CA, USA). A statistically significant correlation between the collected parameters was tested using simple linear regression. Effect sizes were calculated according to Cohen [33]. An *r* value of < 0.10 was regarded as weak, < 0.30 as intermediate and > 0.50 as strong effect. Data collection and statistical analysis were carried out by one of the authors of this study.

## Results

### Patient collective

Overall, 1594 patients that were surgically treated because of an isolated or combined orbital fracture in the years between 2007 and 2017 were included in the study. Patients median age was 42.5 years (SD = 20.8 years), 37.5% of patients were in the age between 15 and 35 years. Younger patient age correlated positively with smaller defect sizes (*p* ≤ 0.001; *r* = 0.106; *n* = 1198). Four-hundred-forty-four patients were female (28%) while 1150 patients were male (72%). There was a positive correlation between the male gender (*p* ≤ 0.001; *r* = 0.34; *n* = 1594) and a younger patient age (*p* ≤ 0.001; *r* = 0.34; *n* = 1594) and physical assault as the cause of injury. Older patient age strongly correlated with accident or falls as the cause of injury (*p* ≤ 0.001; *r* = 0.58; *n* = 1594). The most common causes of injury were physical assault (32.0%, *n* = 511), followed by falls (30.8%, *n* = 491), traffic accidents (17.0%, *n* = 272), sport injuries (6.5%, *n* = 104), injuries during work (5.3%, *n* = 85) and polytrauma (1.8%, *n* = 29).

### Preoperative clinic

In 74.2% (*n* = 1182) of patients, no preoperative symptoms (excluding pain) were recorded. Most often hypoesthesia was described by 13% (*n* = 208) of patients, followed by diplopia (7%, *n* = 112), anisocoria (2.3%, *n* = 36) and visual impairment of the affected eye (1.2%, *n* = 19). Eleven patients were admitted to the clinic with a retrobulbar hematoma (0.7%, *n* = 11) and six patients (0.4%) presented an exophthalmos. Combined fractures patients correlated positively with the occurrence of preoperative symptoms (*p* = 0.003; *r* = 0.13; *n* = 416) and patients that had symptoms prior to surgery also were more likely to have postoperative complications (*p* = 0.011; *r* = 0.29; *n* = 75).

### Fracture morphology

Sixty-two percent (*n* = 987) of patients presented orbital fractures combined with other facial fractures while 38% (*n* = 601) presented isolated orbital fractures. In six cases, it was not possible to collect data due to a lack of documentation. Positive correlations between the occurrence of combined fractures and traffic accidents as a cause of injury (*p* ≤ 0.001; *r* = 0.1914; *n* = 1486) as well as with male gender (*p* = 0.018; *r* = 0.05948; *n* = 1486) could be observed. In 40% (*n* = 630) of fractures a prolapse of orbital soft tissue occurred. The possibility of a prolapse and entrapment of soft tissue increased with the occurrence of a combined fracture (*p* ≤ 0.001; *r* = 0.32; *n* = 1495). The average defect size was 297.8 mm^2^ (SD = 190.8 mm^2^, min = 5.47mm^2^, max = 1500mm^2^). The prolapse of soft tissue occurred more frequently as the defect size increased (*p* ≤ 0.001; *r* = 0.1642; *n* = 1123) and showed a positive correlation with the period between the accident event and the operation (*p* = 0.026; *r* = 0.06613; *n* = 1135).

### Operative procedure

On average, surgery was performed 6.4 days after the injury. With increasing patient age, a longer time between injury and surgical treatment was observed (*p* ≤ 0.001; *r* = 0.1369; *n* = 1469), which was mostly related to existing co-morbidities in elder patients. Post-operative complications were more likely when the time between injury and surgical treatment increased (*p* = 0.007; *r* = 0.07102; *n* = 1458).

The trans-conjunctival incision was (83%, *n* = 1323) the most commonly used approach to the orbit, followed by the infra-orbital incision (5.5%, *n* = 83), which was mainly used in more severe cases and the medial eyebrow incision (5%, *n* = 79). In twenty-nine patients (1.8%), an already existing wound was used for an incision. In 43 patients (2.7%), it was not possible to determine which type of incision had been selected due to a lack of data. The infra-orbital approach was significantly chosen more often with increasing patient age (*p* ≤ 0.001; *r* = 0.1698; *n* = 1551).

### Reconstruction Material.

In 38.3% (*n* = 611), the 0.15 mm PDS^®^ membrane was used to reconstruct the orbital floor defects, followed by the 0.25 mm PDS^®^ membrane (36.2%, *n* = 577) and the titanium mesh (11.5%, *n* = 184). The 0.5 mm PDS^®^ membrane was only used in forty-five patients (2.8%). In 2.6% (*n* = 41) a combination of titanium mesh and PDS^®^ membrane was used. In 5.1% of cases, no data were recorded because of missing documentation. On average, the 0.15 mm PDS^®^ membrane was used for a mean defect size of 225.17 mm^2^ (min = 31.5 mm^2^, max = 750.0, SD = 151.5 mm^2^), the 0.25 mm PDS^®^ membrane for 278.5 mm^2^ (min = 44.6 mm^2^, max = 1019.0 mm^2^, SD = 157.6 mm^2^) and the 0.5 mm PDS^®^ membrane for 282.0 mm^2^ (min = 68.3 mm^2^, max = 750.0 mm^2^, SD = 182.2 mm^2^). The average defect size for a titanium mesh was 455.2 mm^2^ (min = 64.7 mm^2^, max = 1371.2 mm^2^, SD = 196.0 mm^2^). The use of the 0.15 mm PDS^®^ membrane correlated with significantly less postoperative complications (*p* < 0.001; *r* = 0.1932; *n* = 1492), in contrast, the use of a titanium mesh correlated with more postoperative complications (*p* < 0.001; *r* = 0.1763; *n* = 1492). With increasing defect size, the use of the 0.25 mm PDS^®^ membrane and the titanium mesh increased (*p* < 0.001; *r* = 0.4015; *n* = 1135).

### Postoperative complications.

In total, 18% (*n* = 296) of patients presented post-operative complications (Table 1). Most frequently patients presented post-operative hypesthesia (12.9%, *n* = 206), diplopia (4.1%, *n* = 66), followed by intra-orbital hematoma (4.0%, *n* = 62) and displacement of the used reconstruction material (2.1%, *n* = 34). Twenty-five patients with intra-orbital hematomas required drainage and underwent a second operation. Furthermore, twenty patients (1.2%) presented an exophthalmos and eleven patients (0.6%) presented a permanently reduced eyesight after surgery. With increasing defect size, a post-operative complication was more likely (*p* < 0.001; *r* = 0.1703; *n* = 1187). The infra-orbital incision showed a higher risk of post-operative complications (*p* = 0.002; *r* = 0.0761; *n* = 305), such as reduced vision (*p* = 0.028; *r* = 0.05511; *n* = 305) and retrobulbar hematoma (*p* = 0.009; *r* = 0.06531; *n* = 305). Moreover, thirty-six patients (2.2%) developed other post-operative complications that were not directly related to the surgery, such as urinary tract infections, pneumonia or hypertensive crisis.

**Table 1:** Results of the statistical analysis between the collected parameters

Parameter	Age	Gender	Defect size	Type of accident	Fracture morphology	Postoperative complications	Reconstruction material	Time between accident and surgery	Type of incision
Age			** < 0.001**	** < 0.001**	0.29	** < 0.001**	**0.025**	** < 0.001**	** < 0.001**
Gender			0.131	** < 0.001**	**0.018**				
Defect size	** < 0.001**	0.131		0.365	0.056	** < 0.001**	** < 0.001**	**0.026**	
Type of accident	** < 0.001**	** < 0.001**	0.365		** < 0.001**				
Fracture morphology	0.29	**0.018**	0.056	** < 0.001**		**0.021**	** < 0.001**		
Postoperative complications	** < 0.001**		** < 0.001**		**0.021**		0.059	0.618	**0.0014**
Reconstruction material	**0.025**		** < 0.001**		** < 0.001**	0.059			
Time between accident and surgery	** < 0.001**		**0.026**			0.618			
Type of incision	** < 0.001**					**0.0014**			

*P* values < 0.05 were regarded as statistically significant

Bold headings are used for significant correlations

## Discussion

This retrospective study was conducted on 1594 patients who had been operatively treated for orbital floor fractures between 2007 and 2017 at the Department of Oral and Maxillofacial and facial plastic Surgery of the Goethe University Hospital in Frankfurt (Germany). The primary goal of this study was to evaluate which reconstruction material would be best suited for which type and size of orbital floor fracture. A secondary goal was to analyze and discuss the existing data with regard to epidemiological aspects, surgical treatment options and postoperative complications with the current literature.

In the course of the demographic change in most western societies and the increasing number of multimorbid patients, a rigorous patient selection and indication for surgical treatment become more important. Factors, such as age and diabetes mellitus [34, 35], cardio-pulmonary disease [36] and anti-coagulant medication, can significantly increase the risk of post-operative complications and nosocomial infections [37]. In our patient collectively, thirty-six patients (2.2%) developed post-operative symptoms associated with urinary tract infections, pneumonia or hypertension. More than halve of these patients were older than 75 years. Moreover, the risk of domestic falls increases with age [38] which is reflected by the data of our study. Twenty-three percent of our patients were older than 65 years and with patient age the average size of the defect also significantly increased, which could be related to loss of bone elasticity with increasing age. Interestingly, no significant correlation could be found between patient age and post-operative complications in our study which can be an indication for a good patient selection for surgical treatment especially in elder people.

Generally orbital floor fractures can be treated conservatively or operatively. The indication for the latter is still being debated in the present literature. However, it is undisputed that certain symptoms, such as an enophtalmus > 2 mm, a defect size > 1 cm^2^, a severe dislocation of fragments, a limited motility of the bulb with double vision or a prolapse and incarceration of orbital soft tissue must be treated operatively [39–42]. The defect size (> 1 cm^2^) is most often used as an indication for surgery. In our patient collective only fifty-four (3.4%) presented defect sizes < 1 cm^2^ which corresponds to the current recommendations.

The ideal time of surgical intervention is still being debated in the present literature. Burnstine et al. distinguished between an intermediate intervention which should be carried out in case of an early enophthalmos with facial asymmetry with signs of a bulbo-cardial reflex or decreased vision of the affected eye and an early intervention within the first 14 days which should be carried out in case of significant lowering of the affected eye, hypoesthesia of the infraorbital nerve, defect sizes > 1 cm^2^ and double-vision with radiological signs of soft tissue incarceration [43]. This recommendation is also reflected in the present guidelines of the German Society of Oral and Maxillofacial Surgery [44]. Early intervention is recommended by most authors since it is associated with significantly less post-operative complications, such as diplopia or enophthalmos [42, 44–48]. Nevertheless, the decline of soft tissue swelling which usually takes place within five to seven days should be awaited. In the present study, the average time between trauma and operation was 6.46 days (SD = 11). Interestingly, we could observe a significant correlation between the time between trauma and operation and post-operative complications which support the current literature and guidelines that recommend an early intervention. A possible explanation for this correlation could be, that older patients in our cohort were operated significantly later after trauma due to co-morbidities and these patients also had significantly bigger defect sizes, which can result in more post-operative complications.

The vast majority (77.5%, *n* = 1236) of our patients was treated with PDS^®^ membranes (0.15 mm; *n* = 614; 0.25 mm; *n* = 577; 0.5 mm; *n* = 45). Hiding et al. were the first to postulate certain requirements for the ideal reconstruction material like easy handling, good plasticity, natural resorption and sufficient stability and found that PDS^®^ membranes meet most of these criteria. They also found that the degradation of PDS^®^ membranes is associated with an inflammatory reaction and the development a scar tissue that stabilizes the defect after complete resorption within six months [49]. Most authors recommend the use of a PDS membrane only up to a defect size of 200 mm^2^ and argue that the scar tissue could be insufficient in stabilizing bigger defects which could result in more post-operative complications [30, 39]. In our study, the average defect sizes for 0.15 mm and 0.25 mm PDS membranes were 225,17 mm^2^ and 278.5 mm^2^, respectively, which surpasses the general recommendations. The biggest defect that was treated with a 0.25 mm PDS membrane measured more than 1019 mm^2^ and 750 mm^2^ for a 0.5 mm PDS membrane. However, none of those patients presented post-operative complications. Since the reconstruction with the 0.15 mm membrane was associated with significantly less post-operative complications (*p* ≤ 0.001; *r* = 0.1932; *n* = 1492) it can be recommended as alternative for orbital floor defects beyond the 2 cm^2^ which can be found in the current literature.

For bigger (> 300 mm^2^) or instable fractures that include the medial or lateral orbital wall, titanium meshes are generally recommended in the literature [24, 42, 50]. Their rigidity makes them ideal for more complex fractures, and today, pre-bent or CAD-CAM individualized titanium meshes are easy to insert [51]. In our study, 184 patients (11%) were reconstructed using a titanium mesh. Ellis and Tan were the first to show that titanium meshes are an accurate material for orbital reconstruction compared to bone grafts and present a high bio-compatibility to bone tissue [24]. However, there are studies and case reports that associate titanium with hyper sensory and even allergic reactions [52]. Sugar et al. (1992) listed foreign body reactions, complications when inserting or explanting and frequent infections as some of the disadvantages of titanium meshes. We also could observe significantly more post-operative complications in patients that were treated with a titanium mesh compared to the patients that were reconstructed using a PDS^®^ membrane. However, it has to be taken into account that titanium meshes were more often used for bigger and instable fractures (median 421.6 mm^2^) and defect size significantly correlated with post-operative complications. Although not implemented in our patients collective, an additional alloplastic implant that requires consideration for the treatment of large defects, is porous high-density polyethylene (Medpor) [53]. However, this material has also been associated to complications [54]. Randomized and controlled studies that investigate the use of different reconstruction materials for equal size defects are needed, to investigate whether complications are related to the reconstruction material or to the size of the defect alone.

The surgical incision should allow a sufficient overview of the operation area without leaving functional and esthetic impairment [42]. The trans-conjunctival incision was by far (83%, *n* = 1323) the most commonly used approach to the orbit in the present study. Critics of this approach say that it is associated with a reduced overview of the operation area and that it leads to more complications, such as lower eye lid ulceration, entropium and epiphora [55]. In the present study, the complication rate associated with the trans-conjunctival approach was 0.4% and therefore regarded as very low, which is in line with previous studies that also found low complications rates and recommend this approach especially for younger patients because of the ideal esthetic results [56, 57]. However, it should be noted that complications, such as entropium and epiphora, must be regarded as long-term complications and therefore might not be detected because of the three-month clinical follow-up. The infra-orbital incision was rarely used (5.5%, *n* = 83) in the present study because of the visible post-operative scar even though there are studies that report an equal esthetic outcome of the this approach compared to the transconjunctival approach [55]. However, the infra-orbital incision showed a significantly higher risk of post-operative complications, even though the effect was rather weak, so that we would recommend this approach only for emergency treatment where a wide overview of the operation area has to established in short time.

In the present study, a complication rate of 18% was found. In the literature, an interval of complication rates between 1.8% and 44% is given [14, 58–60]. Consequently, a comparatively low rate could be observed in the present study. Moreover, the definition of a post-operative complication in the literature differ widely from severe complications, such as a retrobulbar hematoma to frequent complications, such as post-operative hypesthesia, which explains the wide the range of complication rates published. In this study, the occurrence of post-operative hypesthesia was the most common postoperative complication with 206 cases (12.9%) which is comparatively low to the incidence that is reported in the literature with an interval between 5 and 33% [47, 50]. It should be mentioned that the hypoesthesia specified here was observed and documented during the clinical follow-up within three months. In the literature, depending on the injury pattern, regeneration of neural tissue is generally only expected after 2 weeks to 6 months [61] which permits the assumption that some of these cases a restitutio ad integrum could be expected.

Within the patient collective, a prolapse of the peribulbar soft tissue into the maxillary sinus was observed in 40% of the cases (*n* = 630). On the one hand, this can indicate a “blow-out” fracture in which the volume of the orbital funnel is increased. On the other hand, a prolapse, which is shown as a “hanging drop” in the CT scan, can also be caused by an incarceration of fat or muscle tissue. Independent of the mechanisms, the results show that enophthalmos can appear until days or weeks after the accident or treatment. This is because swelling or a developed hematoma can hide a decrease in volume of the orbital funnel.

In this study, too, corresponding to existing results, a preoperative retro- or infraorbital hematoma in 11 cases (0.7%) and an exophthalmos in six cases (0.4%) could be observed, which are rare spontaneous symptoms after trauma. All six patients with an exophthalmos reported reduced or lost visual acuity. These were due to an increase in pressure of the orbit. The symptom of a retro- / infraorbital hematoma with the formation of an exophthalmos as well as an accompanying change in visual acuity is an emergency indication for lateral canthotomy. Each of these patients was therefore treated with this procedure according to lege artis.

In six cases (9.5%), this corresponds to 0.4% in the entire group, the revision was due to a postoperative visual impairment or visual acuity loss. Permanent blindness was observed in one case. However, this was due to a direct trauma to the bulb and consecutively the visual nerve and not related to the performed surgery. This corresponds to a share of 0.06% in the entire patient collective. In the literature, the postoperative complication of blindness is not consistently stated; a complication rate of 0.04 to 8.3% is reported. [62–64]

The data analysis showed that a revision, which was indicated due to an incorrect reconstruction, occurred significantly more frequently with increasing defect size (*p* = 0.003). In these cases, it could often be determined on the basis of the operation report that the reconstruction of the orbital floor or the insertion of the implant was problematic. Day et al. showed in 2018 that with the help of custom-made titanium mesh, a reduction in complication rates is possible, also with regard to position correction [65]. In the present study, too, an individual CAD/CAM technology-supported production of a titanium mesh was implanted in one patient. The defect was measured with a size of 260.83 mm^2^ and its extent was mainly posterior. However, due to the limitation of the results from one individual case, a general recommendation cannot be made.

Within our data analysis, a correlation between the preoperative and postoperative diplopia was observed. Orbital fractures can be regarded as one of several conditions in the development of a diplopia [55]. Therefore, the probability of double-image perception occurring increases with increasing defect size [48]. On the other hand, a significant correlation between the presence of preoperative symptoms of diplopia and the postoperative occurrence of double vision (*p* = 0.012) could be found. Ramphul and Hoffmann published a review in 2017 in which they examined the same relationship. As a result, they stated that no significance could be determined between the pre- and post-operative occurrences of diplopia. Due to the different etiologies of diplopia, in their opinion, different courses are possible. In this way, a diplopia can be completely eliminated, reduced, intensified or induced by the operation, whereby a statistical normal distribution is generated [66].

A remaining limitation of this study is the retrospective, mono centric design of the study, which may restrict the interpretation of the data. However, this disadvantage is compensated by the highest number of patients with orbital floor fractures evaluated in the literature so far.

## Conclusion

Since the reconstruction of orbital wall fractures with polydioxanone sheets leads to significantly less post-operative complications (especially 0.15 mm polydioxanone sheets) even for defects beyond the recommended 200 mm^2^, they can be preferably recommended for orbital floor reconstruction. Reconstruction with the titanium mesh correlated with significantly more post-operative complications which limit its indication only to large and instable fractures that include multiple orbital walls. The present study could also show that a higher patient age does not necessarily correlate with more post-operative complications providing a strict indication for surgery. Moreover, we could confirm that an early surgery within the first seven days after trauma presents an ideal timeframe and that the trans-conjunctival incision led to significantly less post-operative complications compared to the infraorbital incision and should therefore be the preferred approach.

## Data Availability

Data is available upon reasonable request.
